# Listening in the dark: why we need stories of people living with severe and enduring anorexia nervosa

**DOI:** 10.1186/s40337-016-0117-z

**Published:** 2016-12-15

**Authors:** Janet Conti, Paul Rhodes, Heather Adams

**Affiliations:** 1 School of Psychology, Western Sydney University, Sydney, Australia; 2 Clinical Psychology Unit, University of Sydney, Sydney, Australia; 3 Trauma and Change Research Group, Fort Wayne, IL USA

**Keywords:** Severe and enduring anorexia nervosa, SE-AN, Narrative inquiry, Metaphor

## Abstract

A bold step forward in our approach to Severe and Enduring Anorexia Nervosa invites new paradigms for research and practice. It provides an opportunity for us to explore fault lines, both in our communities of practice and the social structures that inform them. This paper serves to question the medical metaphors on which treatment has been based, in favour of alternative perspectives that resonate more clearly with the lived experience of those for whom it has failed. We invite the consideration of alternative metaphors, which can disrupt the notion of heroic patients (and therapists), mediate against acts of self-silencing and sensitising us to more radical acts of listening. Beyond the randomised trials and manuals it is time for us to listen to the realities of suffering, the minutiae of resistance and the life that can still be lived.

The first session of family-based treatment for anorexia nervosa is called The Intense Scene whereby the therapist empathises with what the family has been through at the hands of anorexia, counteracts feeling of guilt and blame and then discusses what the future might entail if the anorexia is able to continue its domination of their child in the long term [1]. This session is simultaneously compassionate and anxiety-provoking, aiming to bring the family together and bear witness to their pain and stir them to action based on the harsh realities of what the future could hold. Cognitive and gynaecological abnormalities, bone and brain problems and even death. Nothing is left off the table.

Sadly, there is no hyperbole in this session. Parents engaging in family-based treatment have about a 70% chance of bringing about weight restoration in their child [2]. While little is known about the alleviation of psychological symptoms [3], and much less about the personal journey of young people in this form of treatment, weight restoration is still likely to be maintained five years after the completion of therapy sessions [4]. If this treatment fails, however, the prospects of recovery in adulthood are less promising. As the family becomes more exhausted and the young person becomes developmentally and socially dislocated, a return to normal life becomes more and more difficult to achieve.

The next port of call, in terms of formal treatment options, is likely to be cognitive behaviour therapy [5], which will focus on the way you think about food and your shape and weight and seeks to develop your motivation to change. Here, if you have a BMI of 15 or above, there is a 60% chance that you will stick to treatment rather than stop early, possibly due to the need for hospitalisation. You then have a 60% chance of gaining weight to a reasonably healthy range and this will be matched by improvements in the way you think [6].

If you fall through these cracks here, however, this is where you get into very serious trouble. By now, if you have been sick for more than seven years there are no treatments available to you with good scientific evidence [7]. Neurological changes will have set in and your lifestyle, social life and emotional development are likely to be severely truncated. Any memories of a life free of anorexia are beginning to dim.

These trajectories are predicted and marked out by the medical model. Embedded in this medical model is a clinical master narrative that dualistically separates anorexia nervosa as an illness from its antithetical state of recovery, whereby the person returns to some pre-morbid state of normality [8]. Within this context, medical definitions of anorexia are reified and thereby assumed to *be* a person’s reality, rather than one of a number of possible versions of that reality [9].

## Narrative and metaphor

Absent from this clinical-medical storyline are the various ways people understand their experiences and how they integrate these into their broader life story. Analyzing written accounts and personal interviews, researchers have identified various pathways through and beyond anorexia. Unlike the clinical stories, where the central theme is weight restoration, these personal accounts center on healing, hope and the championing of human spirit in the face of adversity [10–14]. Individuals moving through anorexia and into recovery emphasize the processes of reconnecting, discovering or rebuilding a previously neglected or lost part of the self [12, 15, 16]. Given the embedded nature of all experiences, it is not surprising that these changes in self are often accompanied by (re)connecting with others, one’s past, the environment and spirituality, often through re-engaging in meaningful activities [14, 16, 17]. These themes have been drawn from research methods aligned with narrative inquiry [18], whereby the experiencing person makes sense of their life events through use of narrative devices, including metaphor. Metaphors are foundational strategies in personal story-telling that emerge from and shape our understanding of people, objects, and events [19]. A wide variety of such metaphors can be found in the literature related to recovery from anorexia nervosa.

A rite of passage metaphor can provide coherence when a person faces death, choses instead to live and then embarks on a process of self-transformation [16]. Catherine Garrett [16] draws on this metaphor to depict anorexia as liminality*,* as “betwixt and between” ([20] p.95), where the person is no longer who they once were and not yet who they will become. As a person moves to reincorporate themselves into the community they reconnect with themselves (through body, mind and spirit), others and nature. ‘Anorexia nervosa’ becomes a “quest for meaning”, which for some is experienced as a spiritual quest or a “religious conversion experience” with the accompanying sense of “joy, peace and certainty, following a period of despair and suffering” ([13] p. 187). Here, the suffering of anorexia becomes a drive, a force, a necessity of personal transformation, remaking and empowering the sufferer.

The rite of passage metaphor finds theoretical and empirical form in the posttraumatic growth (PTG) model [21]. Here, an adverse event threatens one or more central ideas about the self and the world as a whole, producing cognitive and emotional instability. As the individual negotiates the meaning of the negative event and how to integrate it into their coherent life narrative [21–24], they disengage from the now inadequate pre-trauma idea, while simultaneously constructing a revised sense of self or beliefs about the world that is more congruent with their new reality [21, 22]. Women who recount chronic illness PTG narratives describe themselves as more compassionate, peaceful, and display an increased capacity for accomplishing life goals [22]. Their stories of transformation and growth are not in spite of their illness, but because of illness-related suffering. For example, in Adam’s [22] research into the experience of chronic illness, the participant Linda who lives with chronic lupus explained, “…if you have something that has challenged you and you have dealt with it… It makes you grow inside” (p. 125).

On the other hand, a battle metaphor constructs anorexia as an “overpowering adversary” and the person’s narrative takes the form of “self as hero” ([25] p. 94) who stands up to, battles and vanquishes anorexia from their lives [26]. Within this context, anorexia is construed on entirely negative terms, such as “the prison” ([26] p. 29), “you vampire” (p. 161), “a killer on the loose” (p. 110), “a concentration camp” (p. 110), “a murderer” (p. 114) in an effort to alienate the person from the egosyntonic dimensions of the experience [27]. The limitations of such metaphors have been highlighted by Kelly Bemis Vitousek [27] who has argued that dualistically separating the self into “the real self” who is morally elevated above the demonized “anorexic self” obscures the positive aspects of the experience. These positive dimensions have been construed with the antithetical metaphor of anorexia as “friend” rather than “foe” [28, 29]. Within these contexts, anorexia is positioned as a battle to achieve feeling “an inner strength and invulnerability” ([30] p. 560). Silencing a person in relation to expression of these egosyntonic aspects of their experience of anorexia has a number of risks, including alienating the client from the therapist, [31] along with diminishing opportunity to explore ambivalence and develop discrepancies between action/goals and self reflected values, which is a key component of a motivational therapeutic intervention [27, 31, 32]. Frequently articulated alongside these battle metaphors is the antithetical construction of the “real self” who is simultaneously idealized through frequent use of feminine metaphors as well as forcibly overtaken by anorexia (metaphorically construed as a male oppressor) [27]. Ironically it is this sort of dichotomous thinking that is frequently the target of interventions for anorexia nervosa itself [27].

These metaphors permeate the clinical field, shaping our own understandings and therapeutic practices. Adversarial metaphors can easily be found when considering the major treatments used for anorexia nervosa. In the Maudsley model [1], for example, parents are united to stand up to “the anorexia”, conflict avoidance is challenged in the family meal and weight graphs can soar. For cognitive behavior therapists “the eating disorder psychopathology may be likened to a house of cards with the strategy being to identify and remove the key cards that are supporting the eating disorder, thereby bringing down the entire house” ([33] p. 617). The rite of passage metaphor, the adversarial metaphor and the PTG model are modern, scientific reincarnations of the hero’s journey through darkness and challenge into transformation and enlightenment [34]. As such, they may become invaluable guides for many people. But what about the approximately 20% of people initially diagnosed with anorexia, who, after years of treatment, do not emerge from the suffering transformed in some way that is meaningful to themselves and/or others? Who do not, in fact, emerge from anorexia at all? When this occurs the person is positioned medically and clinically as chronic, with an “unrecovered identity” that is constructed as “intrinsically negative” ([35] p. 8). We as therapists and researchers risk contributing further to these problematic identities if we continue to confine our theorizing and therapeutic conversations to this discursive field of heroic quest, battle and contest.

## Severe and enduring anorexia nervosa

Recently, the term Severe and Enduring Anorexia Nervosa (SE-AN) has emerged in the medical-clinical literature [7, 36, 37] to refer to people suffering with the full range of symptomatology for longer than 7 years. Touyz & Hay [37] call for a “bold step” (p. 2), in recognizing the fact that treatment must turn to focus on harm minimization and quality of life, rather than be focused on cure. Similar to long-term psychosis [38], they advocate for a paradigm shift that recognizes the value of habilitation, based on concepts taken from the consumer-based recovery movement [39]. Scholars argue that these new therapies need to isolate and amplify strengths, build social and support networks and engender self-determination [40].

Research in this area is fledgling. One promising randomized control trial has demonstrated the utility of adapted cognitive behavior therapy in meeting these purposes [7]. Another thematic study of the lived experiences of fifteen people living with SE-AN reveals an interesting weaving of isolation and hopelessness with pride in one’s ability to endure with the eating disorder [41]. There are no stories here of adversaries overthrown, or healing, growth, self-enlightenment, or emergence into a new day. Rather, they are stories of continuing within significant suffering under the influence of anorexia. How can we understand their stories? Are these narratives that we as therapists and researchers, families and friends, are open to hearing? What might we learn from them?

## Listening in the darkness

As therapists, clinicians and researchers, adherence to current medical storylines and metaphors of anorexia may lead us to overlook features or choices that are salient in the lives of people living with SE-AN. As proponents of the enlightenment hero’s journey [34], both in ourselves and in the lives of our transformed and recovered patients, we are ill-equipped to respectfully and attentively listen to these stories. We need a different kind of listening; an attention to metaphors that we do not know, plot lines we cannot follow and problems we have not fully recognized. We have come to think of this as *listening in the darkness*, in which stagnation, pain and chaos are considered valid, meaningful and powerful locations in their own right, not merely a stage along the journey to a better place.

These stories may share resemblances with the Underworld (of Greek Mythology) where suffering fades into the background, “surrounded by the waters of forgetfulness” ([42], p. 17). Or perhaps, as we sit quietly and close within the darkness, we may find that the desolate landscape contains unexpected adaptations and overlooked moments of beauty, much like, “night flowering cacti and scuttling scorpions” ([42], p. 4). Skårderud’s [43] work suggests these stories may also contain metaphors derived from food or the body, such as purity, spatiality, control and self-worth. Research with women recovering from anorexia [9, 15] and living with chronic illnesses [22] suggests these stories will employ paradoxical, multi-faceted metaphors. Some of Conti’s [9, 15] participants described anorexia as a “volcano”, “steel trap” and “tiger”, and enacted sophisticated relationships with anorexia through these metaphors, refusing to totalize it in negative terms. For example, the participant Naomi corrected the interviewer’s interpretation of a tiger as purely negative, explaining, “See, you’re seeing the tiger as not a friend. I am not saying it’s not a friend. When you let go of it and stop holding its tail, it is a friend because it’s just in your vision, its sitting there.” [15]. Similarly, in Adam’s [22] research into a group of women’s experiences of living with chronic illness, Linda described her lupus as, “It means something good. It means something bad… I have opened the door on some things in life and …I’ve had to close the door on others…” (p. 125). Elsewhere in her interview, Linda describes this as an ongoing process, “I am still opening and closing that door”. These relational, flexible and nonlinear metaphors evoke a more complex image of these women’s lived experiences. In these stories, recovery did not entail separating off or vanquishing an experience that is totalized as negative or bad [25]. These stories and metaphors [9, 15, 22] challenge us to rethink illness and recovery. Similar challenges may await us in accounts of women living with SE-AN.

These stories of darkness may also include wastelands of numbness with buried volcanoes of rage. Hambrook, Oldershaw, Rimes, Schmidt, Tchanturia, Treasure, Richards and Chandler [44] found that people living with AN are more likely than the general population to present a socially compliant self-image while feeling hostile. This pattern of extreme social compliance may predate AN [45, 46]. Certainly, lessons in repressing anger start early, as girls who express anger are more likely to be rejected by their peers than girls who express sadness [47]. These messages continue into adulthood [48]. Research exploring invalidated anger experiences indicates they remain vivid and powerful beneath efforts to block or repress them [49].

SE-AN stories of treatment, labeled as “failure” in our current lexicon, have the potential to reveal fault lines in our clinical communities and in our society. Indeed, they may be the canary in the coalmine, whose messages we are currently ignoring. One of these messages may be a critical consideration of the types of metaphors used in understanding anorexia and SE-AN. The current clinical-medical metaphors are adversarial, hierarchical and linear. These are characteristics of masculine metaphors, which carry undertones of competition, strife and the binary of victory/failure [19]. Scholnick’s [19] critical consideration of how masculine metaphors have shaped developmental psychology and the opportunities offered by alternative, feminist metaphors, raises serious questions for clinical psychology as well. The quotes above suggest that women recovering from anorexia [9, 15] or living successfully with a chronic illness [22] employ relational metaphors [25, 50] with characteristics markedly different from the clinical-medical fields; metaphors more appropriately described as feminist [19]. Such metaphors draw our attention to flexibility, context and negotiation [19]. Indeed, Skårderud [43] concludes his delineations of food and body metaphors with a caution that “denial of food may have many different and also opposing meanings at the same time.” (p.172). This observation echoes Naomi’s construction of anorexia as a tiger whereby this metaphor is able to handle the contingencies of a complex social reality that is centred upon how she negotiates her ongoing relationship with the tiger rather than its elimination. How might this change our understandings and interventions for SE-AN? For eating disorders in general?

A second message may be a reconsideration of the patterns and consequences of societal norms severely penalizing girls’ and women’s expressions of anger. Walter and LaFreniere [47] point out that this is a gender-based pattern, with boys’ expressions of anger being more likely to be popular. Disturbingly, even when women do express anger, it is more likely to be interpreted as sadness [51]. This hinders a woman’s ability to express herself, while signaling the futility of attempting to express her real emotions. Additionally the repression of anger is significant when its expression may be understood as signaling an identity violation through the disclosure of one’s “cherished values” ([52] pp. 98–99). Like other scholars exploring the role of gender in various psychological disorders, we need to be attentive to how “unworkable gender arrangements” (Bepka & Kristan in [53], p. 205) may impact women living with SE-AN. Do our current therapeutic approaches encourage exploration and expression of anger? Or are we unconsciously and unquestioningly mirroring a culture Lerner describes as “more comfortable with women who feel inadequate, self-defeating, guilty, sick, and diseased, than with women who are angry and confronting.” ([53], p. 203). How can we help our client’s negotiate society’s gendered patterns of emotional expression? What roles can we play in de-gendering these patterns in families and society?

## What role can therapy and research play?

Whether or not we are aware as a profession, we are powerful in selecting the stories and metaphors that become encoded in research and enacted in therapy sessions. The metaphors that we draw upon in our work are not merely descriptive, but also shape our understandings, the ways our clients engage with their life, and the processes of their identity formation. Through our reliance on masculine, linear, adversarial and growth-focused metaphors we have privileged these ways of thinking about illness. Like other forms of engendered thinking, this privilege is the legacy of a particular zeitgeist. It is time to re-examine these metaphors and think critically about our exclusive reliance upon them. Are we, like the 12^th^ century Christian European leaders, producing “dissociation, … disaster” (p.5) through our failure to adjust our stories and explanations to reflect our changing world [54]? Have we been unintentionally harming the very people we have devoted our lives to assist through our reliance on these metaphors? Just as noted mythologist, Joseph Campbell placed responsibility for the emerging “spiritual disaster” ([54] p. 5) of 12^th^ century Christian Europe upon the organizations and individuals entrusted with the cultural myths, so too is it psychology’s responsibility to revise our signs and symbols, so that they speak with and reflect the lived experiences of previously neglected and marginalized people?

These questions may be particularly salient for the lives of women living with SE-AN. The repeated correlations between society-taught self-silencing and anorexia raises challenging questions regarding how we may be participating in practices of silencing when we overlook or ignore patients’ feminist metaphors, and instead guide them to shape their stories along the characteristics of masculine ones. Skårderud’s caution that a misunderstanding of the nature of anorexia can lead therapist to “elicit potentially treatment-destructive interventions” ([43], p. 250) takes on new meaning and possibilities in this light. We need to think critically about how our reliance on absolute medical definitions of recovery, based on diagnostic nosology, can be experienced as acutely oppressive by those living with SE-AN. We also need to recognize that an adversarial approach to anorexia and one that only allows for a heroic counter-adversary can serve much of the same function. As Campbell cautions, individuals for whom “the authorized signs no longer work … [who are] Coerced [into] the social pattern … can only harden to some figure of living death.” ([54], pp. 5–6). Remaining attentive and receptive to feminist characterizations of the journey of those who experience anorexia allows us to explore a greater diversity of goals and ways of living, ones that are contextually appropriate for the person and constructed in collaborative dialogue with them.

Engaging differently with stories of darkness will require a relational approach that takes a position of respectful engagement; one that is not dominated by efforts geared towards growth and change [19, 25]. *Listening in the darkness* will first involve the radical act of ‘not knowing’ [55], where our expertise, both theoretical and practice-based, is abandoned in favor of intersubjectivity. Drawing on various forms of participant action research [56, 57], we propose an attitude and approach where researchers and participants share the power of knowledge generation. Here, researchers voluntarily set aside their elevated position as expert, choosing to work beside and with participants, while acknowledging the unique understandings and insights they bring to the table. Such a paradigm shift requires a willingness to alter entrenched beliefs [56, 57]; to be confused and frustrated and to be quiet when necessary [58]. From this position, we may come to learn about the suffering of the other and bear witness to the life that remains. We must listen to what is “absent but implicit” ([59] p. 35), with the voice of the narrator positioned as central. To counterbalance the historical privilege of professional and medical expertise, and to learn anew, we need to emphasize the ‘insider knowledge’ [60] of women living with SE-AN. The successful outcomes of projects employing this approach with vulnerable populations and issues, such as sobriety among Yup’k [57], Inuit suicide prevention [61] and survivors of domestic violence [58] hold the promise of valuable insights and pathways. Listening to stories of people who are living with SE-AN can be conceptualized as a two-way process, that provides scope for “letting stories breathe” ([62] p. 2) life into the experiencing person. This process holds the possibility of transformation for all involved. Having their thoughts and perspectives valued by others could itself be an empowering experience for women long silenced by society. As we tune our ears and minds to listen to new types of stories, our shifting understandings will shape and change our research and therapy practices. Sharing these stories and insights with our colleagues will provide a pathway to transformation of the field.

If we are, as Touyz & Hay [37] assert, in search of a new paradigm for understanding and treating SE-AN, then perhaps it is time for a paradigm shift in methodology as well. If our models and metaphors embody masculine, linear and fixed ways of viewing SE-AN, further research building off of these ideas will continue to misrepresent or remain blind to alternative possibilities. If we are searching for radical reconceptualisations of SE-AN, then it is time to turn to inductive, qualitative methods of inquiry. They have a rich history of producing powerful new insights, changing our understanding of religion, [63] identity [64], life goals [65] and posttraumatic growth [66]. It is time to conduct studies, firstly, that are phenomenological in nature, emphasizing the experiencing individual and the meanings they construct from their experiences. Here, a woman living with SE-AN can tell her story in her own voice, using her own language, in a manner that best fits her own interaction style. This may be particularly important when sharing emotionally laden events she has long remained silent about [49].

Efforts to listen in the darkness reach beyond the interview itself into the methods of analysis. Narrative analysis, with its focus on how the person chooses to tell their story [67] provides a rigorous approach for putting the previously neglected voices of women living with SE-AN in the center, facilitating our ability to understand how they make sense of their own SE-AN experiences. In this approach, emotions remain experientially complex, unique, and located in specific contexts, supporting exploration of this neglected area of SE-AN. Here, the women become the teachers, and we the students, as we strive to understand their experiences of the world. Discourse and discursive analysis (for example [68, 69]) moves the researcher closer into the participant’s story, exploring metaphors and other linguistic devices the storyteller uses to convey their personal meaning that “can cast light on why we understand ourselves as we do” ([69], p. 107) as well as providing scope for dialogue between the study of discourse and affect and emotion research [70]. As our discussion of metaphors above indicates, the gap between current medical-clinical metaphors and those employed by the women themselves suggests that we have much to learn here. Finally, thematic analysis allows us to generate ideas of how these different metaphors, emotion and experiences interweave into the complexity of a person making sense of their SE-AN. Thematic analysis is a particularly useful method for understanding organizing principles, patterns and interactions both within and across participant interviews [71].

## Conclusions

We need research that is sociologically engaged, exploring those fractures that SE-AN reveals about our cultural context and ourselves. Women’s SE-AN experiences must be embedded within a critical exploration of gender, gender ideology and the possible tyranny of the hero’s story, as they play out in the professional fields these women turn to for help, and the larger society in which they live. Here, at the end of the long road, we might come to acknowledge that women’s bodies and narratives must be defined by themselves, while we acknowledge their unique knowledge and our responsibility to learn from them.
